# Supplementary figures and images for: The CLIC/GEEC pathway regulates particle uptake and formation of the virus-containing compartment (VCC) in HIV-1-infected macrophages
Source: PLoS Pathog. 2025 Mar 11;21(3):e1012564. doi: 10.1371/journal.ppat.1012564 (PMC11925468; doi:10.1371/journal.ppat.1012564)

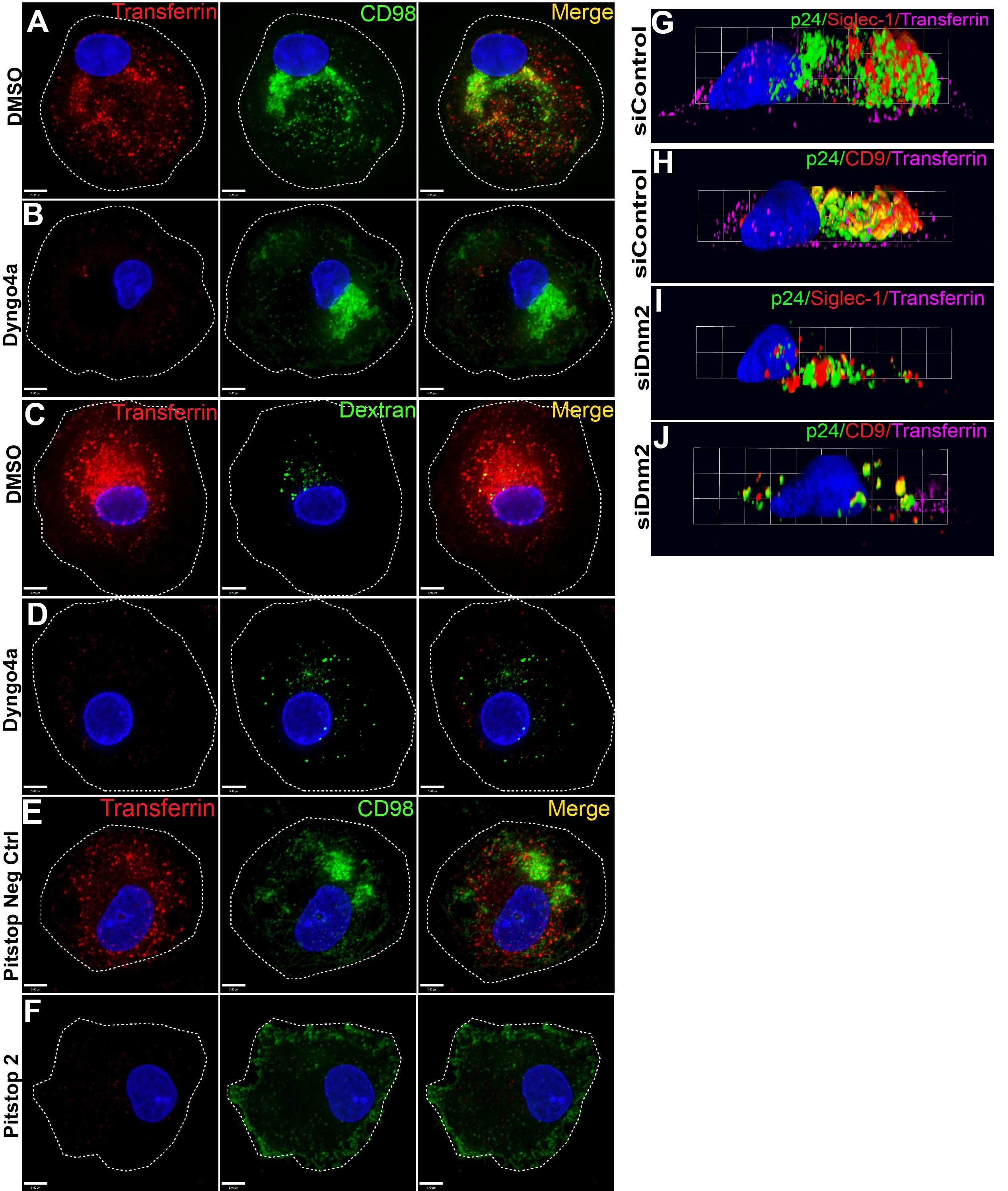

Supplement: S1 Fig — (A-D) MDMs were treated with either 20 μM DMSO or Dyngo4a for 30 minutes. Transferrin and CD98 Ab or Transferrin and 70kD Dextran internalized for 30 minutes before fixation of MDMs. MDMs were washed, fixed in 4% PFA and, and stained with DAPI. (E-F) MDMs were treated with either 20 μM Pitstop negative control or Pitstop 2.0 for 30 minutes. MDMs then internalized transferrin and CD98 Ab for 30 minutes before fixation. Cells were washed, fixed in 4% PFA and, stained with DAPI. Scale bar = 5.40 μm. Data are representative of least three independent experiments. (G-J) 3D side view of the images from Fig 1G-1J. (TIF) [file ppat.1012564.s001.tif]

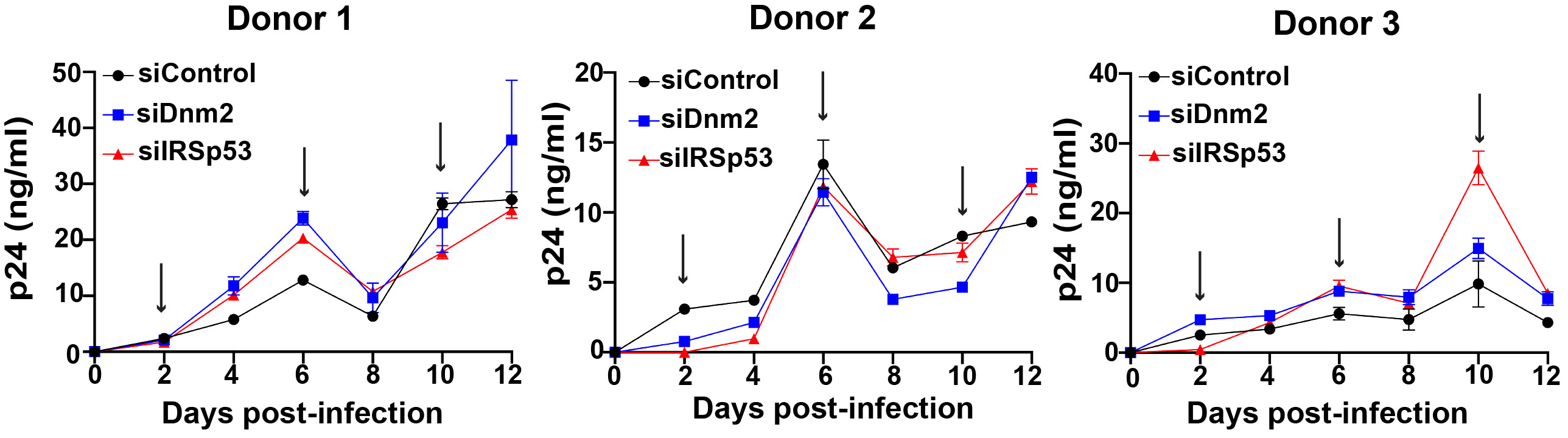

Supplement: S2 Fig — p24 production and release from dynamin-2, IRSp53 or control siRNA treated and HIV-1BaL-infected MDMs was assessed over 12 days using a p24 ELISA. The efficiency of particle production is plotted as mean ± SD of the extracellular p24 over the 12 days of infections from each of the different donor used this experiment. Arrows indicate 3 timepoints when the medium was changed, resulting in a transient drop in the amount of extracellular p24. (TIF) [file ppat.1012564.s002.tif]

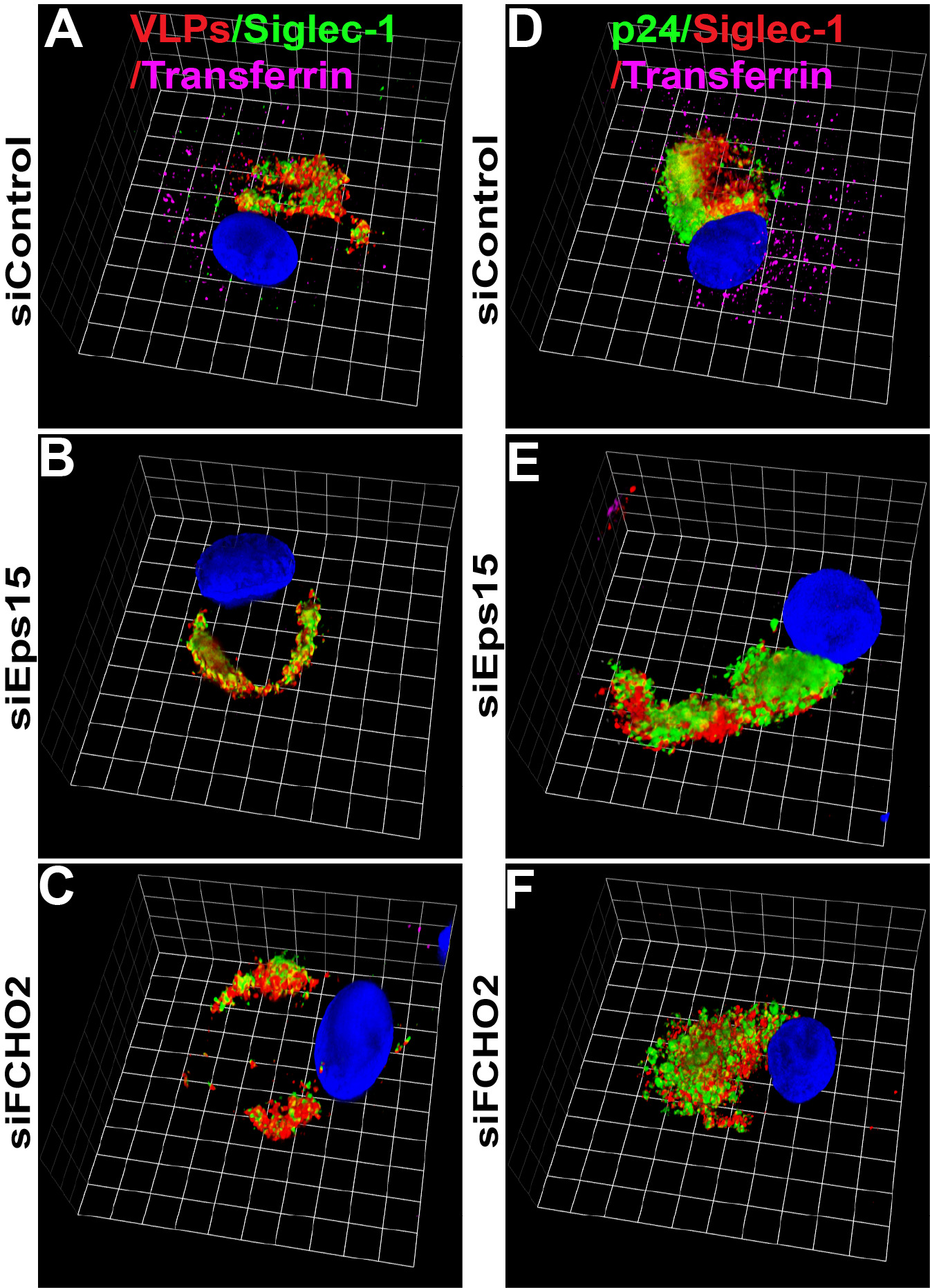

Supplement: S3 Fig — (A-C) 3D view of the images from primary Fig 2 evaluating VLP uptake into VCC following siRNA treatments. S3A corresponds to panel A in Fig 2, S3B corresponds to panel D in Fig 2, and S3C corresponds to panel E in Fig 2. (D-F) 3D view of the images from primary Fig 2 evaluating VCC formation in infected MDMs following siRNA treatments. S3D corresponds to panel H from Fig 2, S3E corresponds to panel I from Fig 2, and S3F corresponds to panel J from Fig 2. (TIF) [file ppat.1012564.s003.tif]

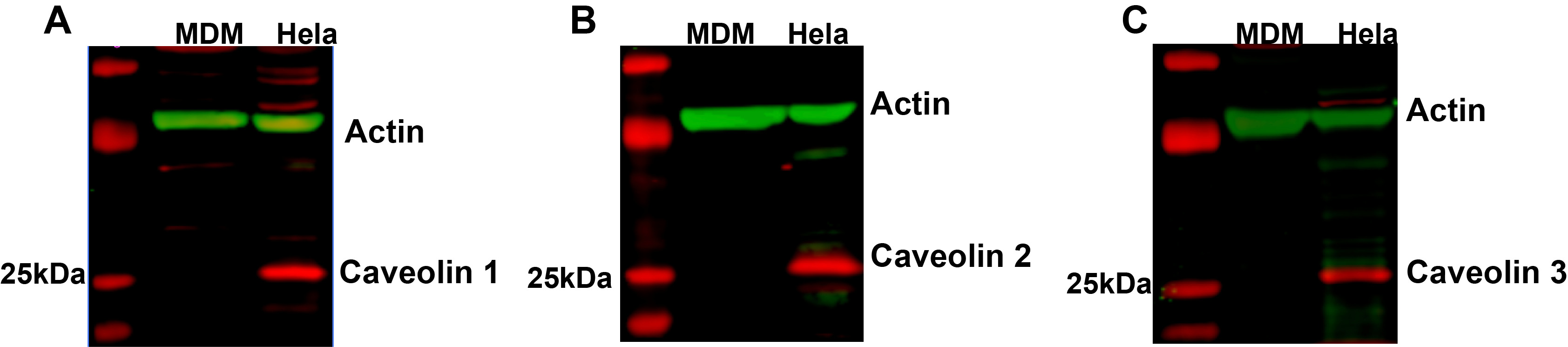

Supplement: S4 Fig — (A) Western blot for caveolin1. (B) Western blot of caveolin 2. (C) Western blot of caveolin 3. (TIF) [file ppat.1012564.s004.tif]

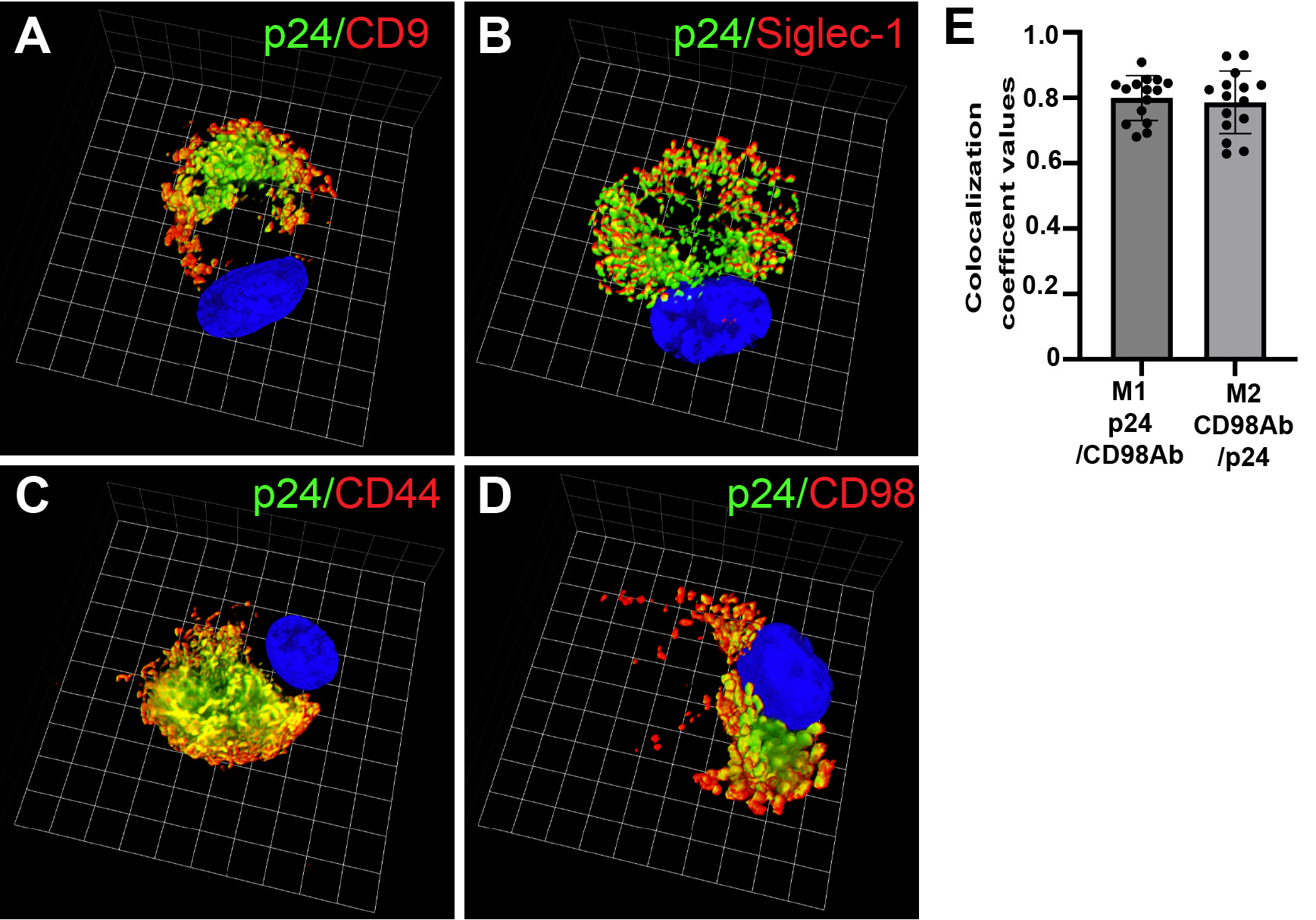

Supplement: S5 Fig — (A-D) 3D view of the images from primary Fig 3A-D. S3A corresponds to Fig 3A, S3B corresponds to Fig 3B, S3C corresponds to Fig 3C, and S3D corresponds to Fig 3D. (E) Colocalization coefficient values for p24/CD98 (M1) and CD98/p24 (M2). (TIF) [file ppat.1012564.s005.tif]

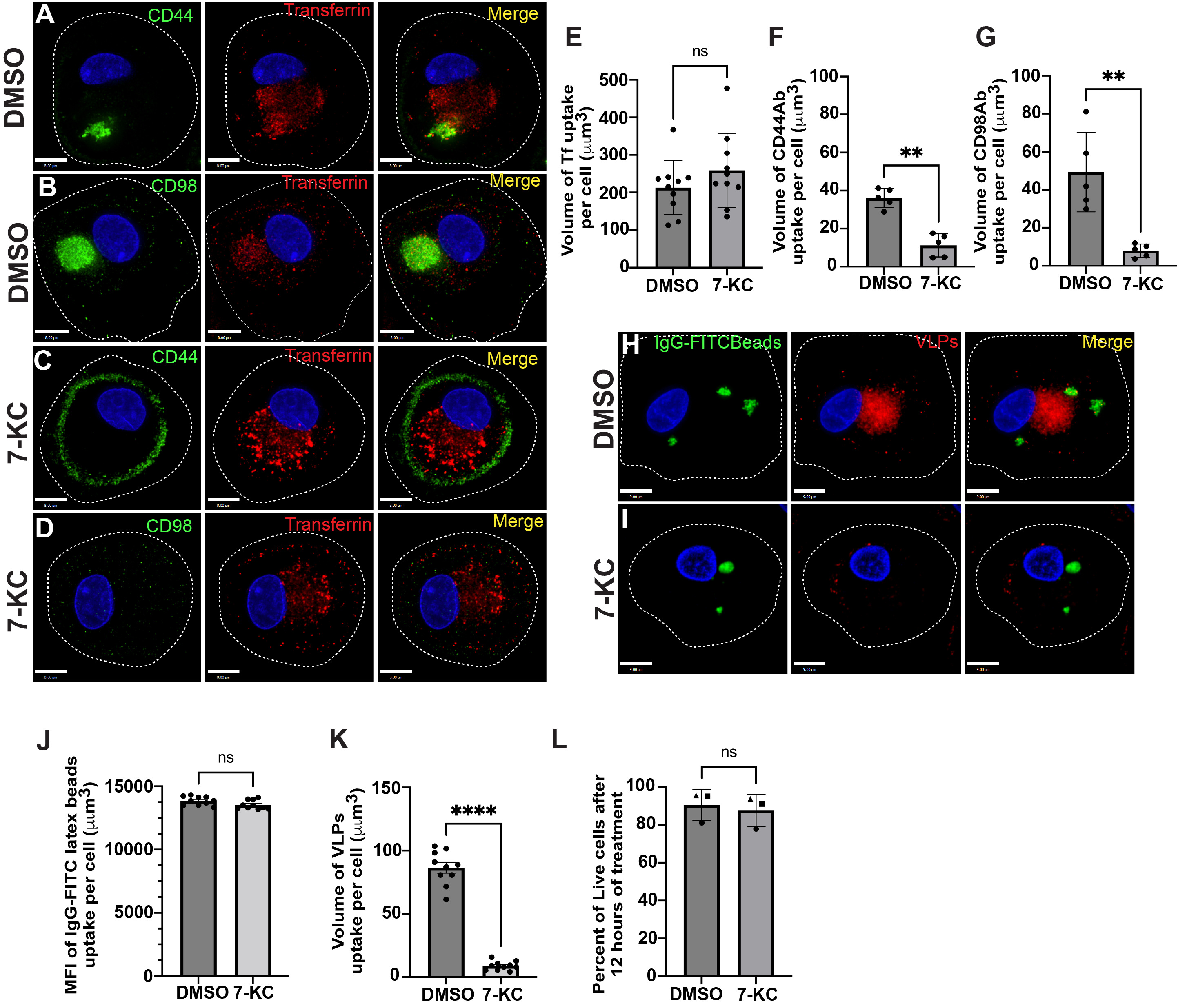

Supplement: S6 Fig — MDMs were treated with 30 μM DMSO (A and B) or 7-KC (C and D) for 12 hours. Transferrin and CD98 Ab or CD44 Ab were added and internalized for 30 minutes before fixation. MDMs were washed, fixed in 4% PFA and stained with DAPI. Scale bars = 8.00 μm. (E) Volume of transferrin uptake per cell (n.s = P value of >0.05 by Mann-Whitney Test). (F) Volume of CD44 Ab uptake per cell (μm3), mean with SD. (G) Volume of CD98 Ab uptake per cell (μm3), mean ± SD. (H and I) MDMs were treated with 30 μM DMSO or 7-KC for 12 hours, followed by addition of mcherry HIV-1 VLPs and incubation for 14 hours. FITC-labeled IgG-opsonized latex beads were internalized for 1 hour. Scale bars= 9.00 μm. (J) MFI ± SD of opsonized beads per cell (n.s = P value of >0.05 by Mann-Whitney test). (K) Volume of VLP uptake per cell with the mean ± SD. (****P value of <0.0001) (Mann-Whitney test). (L) Percentage of live cells after 12 hours of treatment as determined by Zombie live/dead indicator staining (BioLegend) and analyzed via flow cytometry (mean ± SD) (Mann-Whitney test). Data are representative of least three independent experiments. (TIF) [file ppat.1012564.s006.tif]

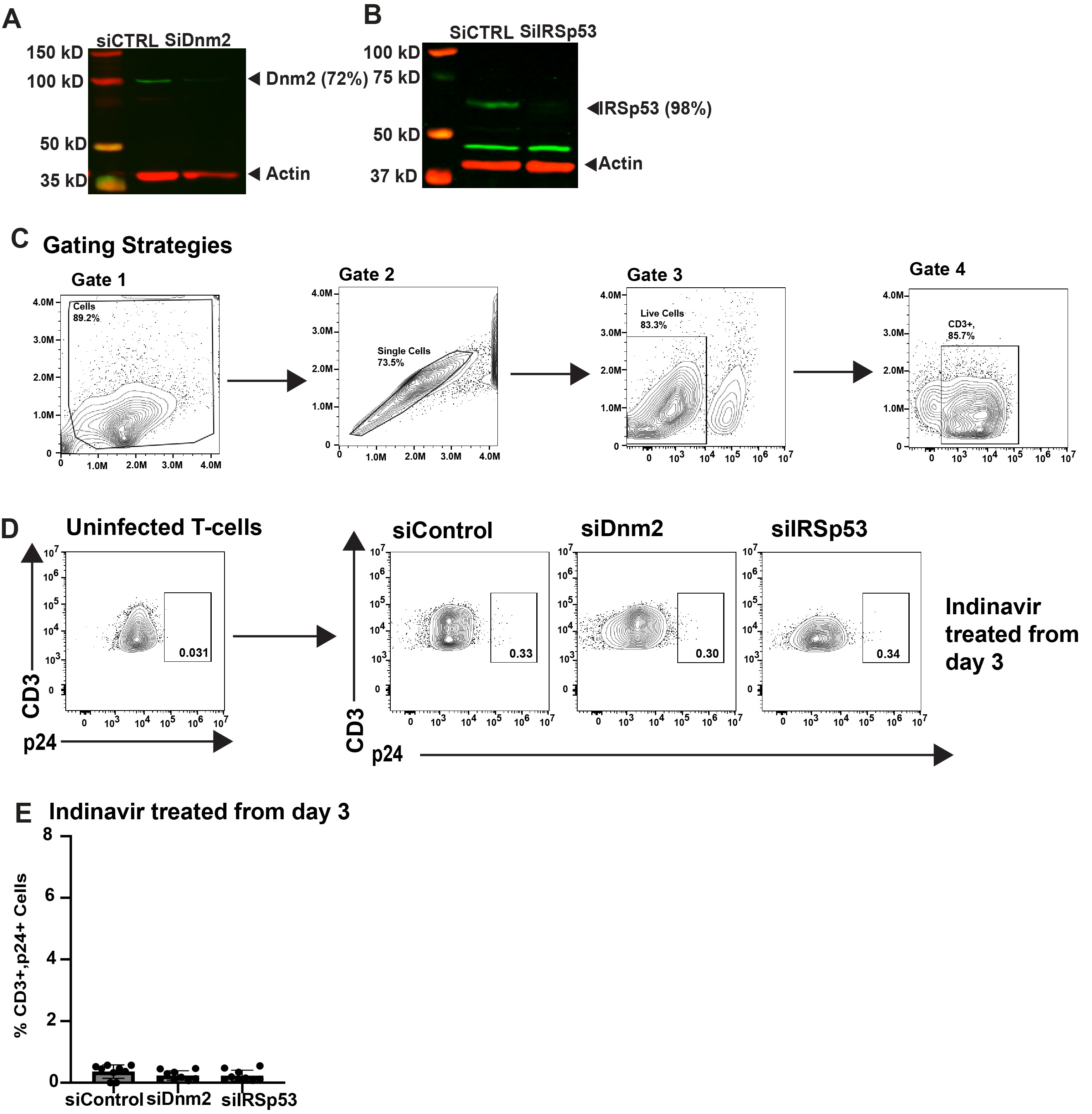

Supplement: S7 Fig — (A) Representative knockdown of Dnm2 in MDMs that were transfected with either siControl or siDnm2, as shown by western blot. (B) Representative knockdown of IRSp53 in MDMs that were transfected with either siControl or siIRSp53, as shown by western blot. (C) The gating strategy used to determent the percent of live CD3+ and p24+ T-cell. (D) Flow cytometry plots gated for CD3+ and p24+ from Donor 2 showing the percent of HIV-1 transmission in T-cells after indinavir starting day 3 post-infection. (E) Bar graph of the mean ± SD of percent of CD3+ p24+ cells, where all MDMs were treated with indinavir from day 3 post-infection. Data from 3 donors, with 3 technical replicates each. (TIF) [file ppat.1012564.s007.tif]

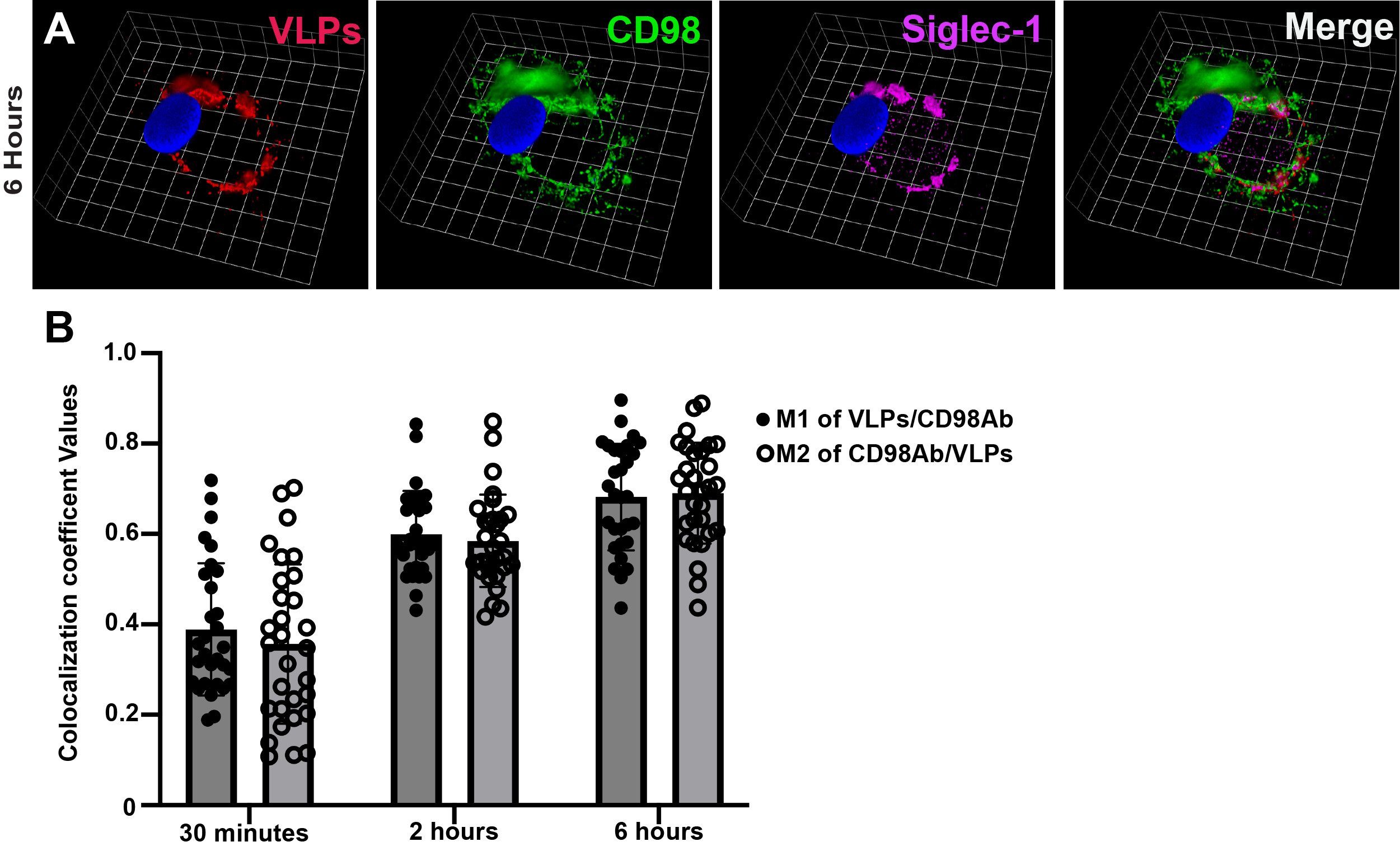

Supplement: S8 Fig — (A) 3D representation of the indicated VCC components from Fig. 7 at 6 hour time point. (B) M1 and M2 colocalization coefficients for VLPs and CD98 antibody over time. (TIF) [file ppat.1012564.s008.tif]

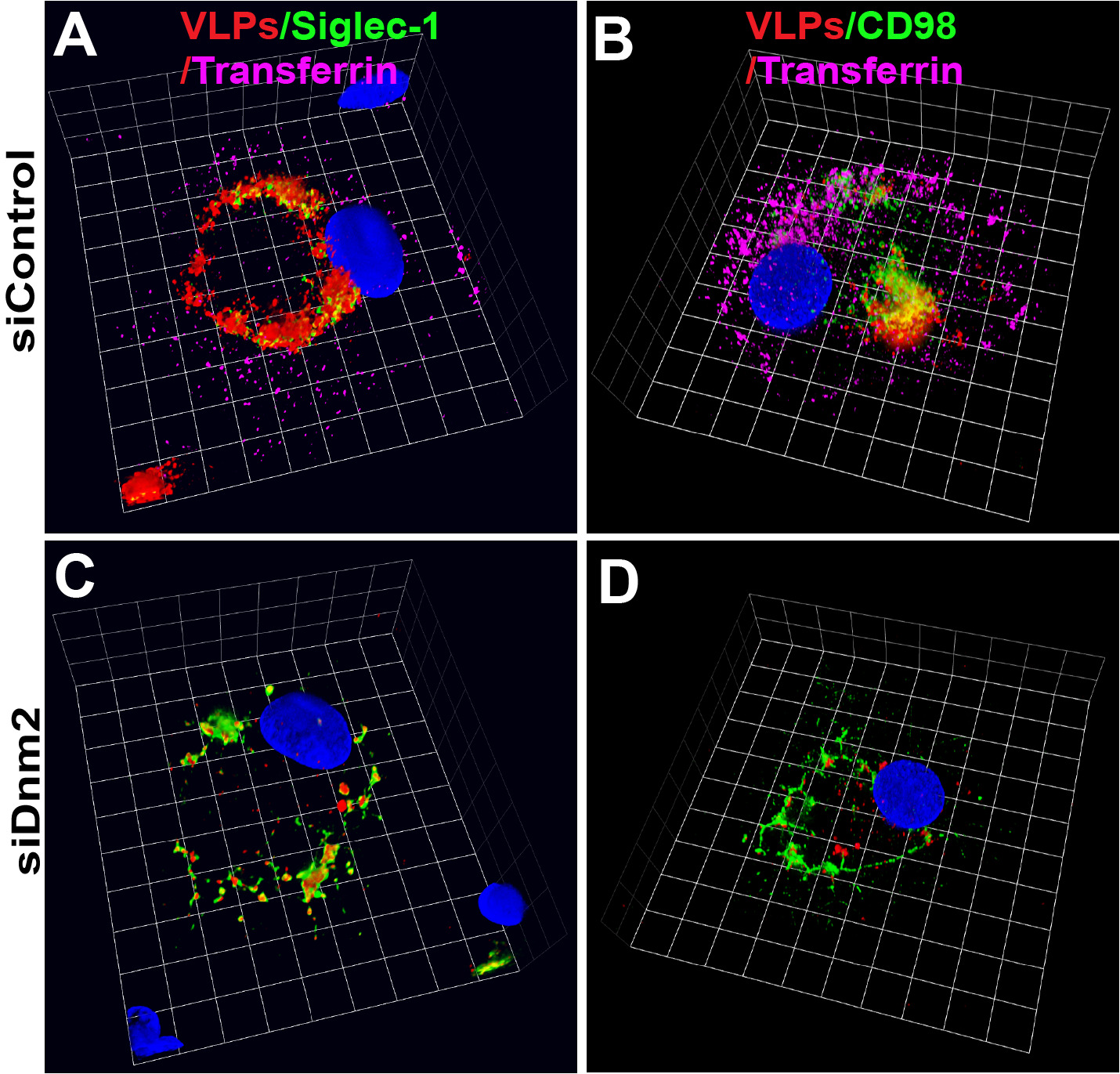

Supplement: S9 Fig — (A) 3D representation of overlay image from Fig 8E. (B) 3D representation from Fig. 8F. (C) 3D representation from Fig. 8G. (D) 3D representation from Fig. 8H. (TIF) [file ppat.1012564.s009.tif]
