# Supplementary material for: The CLIC/GEEC pathway regulates particle uptake and formation of the virus-containing compartment (VCC) in HIV-1-infected macrophages
Source: PLoS Pathog. 2025 Mar 11;21(3):e1012564. doi: 10.1371/journal.ppat.1012564 (PMC11925468; doi:10.1371/journal.ppat.1012564)
Supplement: Attachment — Submitted filename: ResponsetoReviewers2025.pdf [file ppat.1012564.s013.pdf]

Response to Reviewers' Comments

Part I - Summary

Reviewer #1: In this paper, Candor et al. identify novel molecular players involved in the trafficking route of HIV-1 captured via Siglec-1 in macrophages…This is therefore a well conducted study that adds novel findings to the current knowledge of how VCCs are formed in macrophages. <u>The study lacks however trans-infection assays and re-evaluation of some conclusions bearing in mind prior studies.</u>

> **Response: Thank you for this comment. We agree that trans-infection assays to establish the functional relevance of these findings will improve the paper. We spent the past several months concentrating on these assays, and have found consistent results now from multiple donors that establish that 1) knockdown of dynamin-2 significantly reduces transmission of HIV via trans-infection from infected MDMs to autologous primary CD4+ T cells; and 2) knockdown of IRSP53 reduces trans-infection from MDMS to autologous primary CD4+ T cells to an even greater degree than dynamin-2 knockdown. Thus, both of the effects that constitute the major focus of this manuscript show biological/functional relevance in diminishing trans-infection. These data have been compiled into an additional figure, new Figure 6, and are discussed in the revised manuscript.**

Reviewer #2: The authors previously demonstrated the role of Siglec-1 in the formation of virus-containing compartments (VCCs) and HIV-1 transfer from macrophages to T cells in 2017 (PMID: 28129379)…The experiments are mostly well conducted, the paper is well written, follows a coherent logic and the results are convincing. <u>However, this work lacks novel approaches compared with what the authors have previously published before. Overall, this manuscript needs some improvements for publication, especially deeper mechanism(s), different representations of the data (e.g. volume of VCC has to be represented in 3D) and study of the role of CLIC/GEEC pathway in HIV-1 transfer to T cells (from macrophages and potentially from dendritic cells).</u>

> **Response: Thank you for this comment. As outlined above, trans-infection experiments have been the primary focus during our revision, and are now included in Figure 6, showing the significance of dynamin-2 and the CLIC/GEEC pathway in trans-infection. Beyond this major addition, we now include data showing that following dynamin-2 knockdown, CLIC tubules with VLPs and Siglec-1 are formed, but are unable to expand into the deeper, larger compartments that represent VCCs (New Figure 8, represented in 8E-8H). The meaning of this is discussed further in a response to specific criticism below. Additional 3D representations have also been added in Supplemental Figures S1 Fig, S3 Fig, S5 Fig, S8 Fig, and S9 Fig to address this comment/concern.**

Reviewer #3: The Spearman lab previously reported that the so-called virus-containing compartment (VCC) in HIV-infected macrophages is formed through the capture of ganglioside-containing virus-like particles (VLP) by the cell surface lectin Siglec-1… Altogether, the conclusions are significant and supported by the data, the data are clearly presented, and the paper is well-written.

> **Response: We thank the reviewer for this summary comment.**

Part II – Major Issues: Key Experiments Required for Acceptance

Reviewer #1:

1. The authors should carefully consider using a different term than “endocytosis” in the context of this study (including the title), as the VCC they refer to remains attached to the extracellular space and is therefore not formally “endocytosed”.

Response: We have altered the title and in the text have replaced the word “endocytosis” with “uptake” or “internalization” where the wording discusses VLP or HIV particle uptake. We retained the use of the term “endocytosis” when discussing endocytic pathways, such as the CLIC/GEEC pathway, and in some of the Discussion section.

2. While the involvement of Dynamin 2 in VCC formation is clearly shown by the silencing experiments performed in Figure 1, it is less clear to me what is the actual role of this molecule in the formation process. The proposed activity on tubule excision shown in Figure 7C is not supported by the observations presented in this paper and somehow leads to the striking suggestion that scission is required to form a compartment that remains connected to the extracellular space. This aspect needs further experimental validation or clarification.

Response: We agree that we have not shown yet exactly how dynamin-2 contributes to particle uptake and VCC formation. However, we partially addressed this with new data with dynamin-2 knockdown, VLP uptake, and CD98 staining of CLIC tubules in Figure 8E-8L. This figure shows that with knockdown of dynamin-2, Siglec and VLPs are present in small peripheral tubules representing “CLIC” tubules, and these tubules fail to progress to full enlarged membranous compartments representing the VCC. This adds to the evidence that dynamin-2 is important in stages following initial particle uptake. While it does not establish that membrane scission contributed by dynamin 2 is required for the membrane dynamics leading to VCC formation, it is a reasonable hypothesis that can be tested in future work. We added a question mark on the dynamin representation in the graphical representation (now Figure 9), and clearly state in the Discussion that we do not know if membrane scission activity is required, while not abandoning this hypothesis (Discussion, line 600-602).

[More under 2.} Interestingly, prior studies involving DCs identified DNM2 as a key molecule whose knockdown increased HIV-1 uptake but decreased HIV-1 trans-infection, what could be rescued by inhibiting micropinocytosis https://doi.org/10.1016/j.cell.2015.12.036. DNM2 was found to stabilize cortical actin and limit HIV-1 entry…<u>Thus, it would be critical to show the consequences of DNM2 knockdown on micropinocytosis and explore if DNM2 knockdown could interfere on 1) HIV-1 VLP uptake in macrophages, 2) have an impact in micropinocytosis and 3) rescue HIV-1 trans-infection.</u> This could help to depict a model for DNM2 in Figure 7C based on generated evidence.

Response: The Menager paper from the Littman lab that is cited here showed that DNM2 <u>inhibited</u> trans-infection and results in accumulation in what were termed macropinosome-like vesicles (*Figure 5 from the cited paper: Inhibition of DNM2 Function in MDDCs Reduces HIV-1 trans-infection and Concentrates Viral Particles in Macropinosome-like Vesicles*). We thank the reviewer for pointing out these findings, as they are certainly relevant to the current

manuscript, and this is now cited in the revised text. While we were unable to inhibit VLP uptake by chemical inhibition of macropinocytosis, data that we did not include in the manuscript, uptake into vesicular structures is completely consistent with what we show here. To further align our findings with those from the cited paper, we now include Figure 6, showing that dynamin-2 knockdown significantly reduces trans-infection. Furthermore, we now include in Figure 8E-8L additional data showing that VLP uptake was inhibited upon dynamin-2 knockdown. In this figure, VLPs are taken into tubular/vesicular structures at the periphery of MDMs. Therefore, our findings are lartely consistent with those of Menager and Littman, while the nature of the structures as shown in our manuscript is tubular and related to the CLIC/GEEC pathway rather than macropinosomes. This is discussed on lines 602-607 of the Discussion section in the revised text.

3. Given the involvement of DNM2 in VCC formation, is there a role for caveolin in this process?

We carefully considered caveolin's potential role in VCC formation. However, we found that caveolin 1, 2, and 3 are not expressed in MDMs, as shown by RNAseq, qPCR, and by western blotting in our own hands, included in Supplemental Figure S4. This is consistent with what has been reported previously (*Virology 409 (2011) 234–250).*

Can "lipid raft" dependent pathways also influence Siglec-1 mediated internalization of HIV-1 as previously shown for DCs? (https://doi.org/10.1128/jvi.77.23.12865-12874.2003)

This paper shows that HIV particle attachment to DCs was disrupted by removal of cholesterol by methyl beta cyclodextrin or filipin III. We performed preliminary experiments with methyl beta cyclodextrin treatment of MDMs, and found that particle uptake and VCC formation were indeed inhibited, consistent with the paper cited. We prefer not to add these data to the current paper, however, as we did not yet ascertain the specific step of inhibition and rule out effects on overall health of the MDMs, as well as further controls that would allow replication of the paper cited above. We do now cite that paper in the Discussion section of the current manuscript. Since CLIC/GEEC-mediated endocytosis itself occurs in specific raft-like microdomains of the PM, we think that this is entirely consistent, as these somewhat harsh treatments to remove cholesterol would also disrupt this pathway. This is now discussed in lines 609-614 of the Discussion section in the revised manuscript.

4. Figures 3, 6 and 7 lack associated quantification and do not disclose how many experiments are analyzed. These studies should be done at least in 3 independent experiments and quantified as in the rest of the figures of the paper.

Response: We have made sure that each of the Figure legends clarifies that a minimum of 3 independent experiments were performed for each of the findings shown. We have added additional quantitation wherever it was missing.

5. The paper could increase relevance by addressing the impact of inhibiting the CLIC/GEEC pathway in HIV-1 trans-infection from macrophages to bystander target

cells. This is critical to support the concept of interventional strategies aimed at limiting this potential viral reservoir, as suggested by the authors in the discussion section.

Response: See response regarding trans-infection above.

Reviewer #2:

Main comments:
1/ Most of the images are of good quality but should be complemented by z stack (as supplemental movies for example) and 3D reconstructions are necessary, in particular because most of the quantifications rely of VCC volume. The images in 2D are not sufficient for the reader to assess the effects of drugs and siRNA on the 3D volume of VCCs. It needs to be improved in the whole manuscript.

Response: We thank the reviewer for this suggestion. Some but not all of our previous figures included XZ projections or 3D reconstructions to show where in the cell the VLPs/virus and cellular markers were seen. For all major conclusions, where this was not already present in the primary figure, we added 3D representations, currently in Supplemental Figures S1 Fig, S3 Fig, S5 Fig, S8 Fig, and S9 Fig.

2/ It would be more informative (compared to the previous paper from the same team) to go further into the mechanism(s) and to study for example the role of microtubules or transport proteins. The role of GRAF1 mentioned in the discussion could also be tested.

Response: We chose in revision to address primarily 1) the trans-infection experiments and 2) to examine VLP uptake into CLIC tubules following depletion of dynamin-2, and added figures for both of these findings. The data on uptake of VLPs into thin tubular structures together with CD98 (Figure 7E-G) provides some more mechanistic insight into the process.

3/ The role of CLIC/GEEC pathway in HIV-1 transfer to T cells should be tested as a functional assay.

Response: Studies in Figure 6 show that inhibition of CLIC/GEEC-mediated uptake by depletion of IRSP53 inhibits trans-infection of T cells, addressing this concern.

4/ What is happening in Dendritic Cells ? The VCCs in these cells are supposed to be slightly different compared to the ones in macrophages. Whether the described mechanisms of VCC formation are similar in DCs (or not) could be studied.

Response: We agree with this reviewer that examining the role of the CLIC/GEEC pathway in DCs in a comprehensive manner is worth pursuing, but we submit that this is beyond the scope of the current manuscript in revision. Our laboratory focuses on macrophages and microglial cells as model myeloid cells relevant to HIV biology, and we have not previously worked on DC biology. This would require a separate body of work to perform in a satisfactory manner, and we will likely leave this to laboratories focusing on DC biology.

Reviewer #3:

1. It is possible that virus replication over several cycles was inhibited by knocking down dynamin 2 or IRSp53, which in turn could have indirectly affected VCC formation. Indeed, it has been reported that IRSp53 assists in the assembly of HIV-1 particles. For this reason, it appears important that the authors clarify whether virus replication was affected under their conditions.

Response: We measured released virus production by p24 in controls and in cells following dynamin-2 or IRSp53 knockdown. We found that although there was some experimental variation, total p24 production was not significantly altered by these interventions. These data are included in S2 Fig.

2. Although the CLIC/GEEC pathway is typically dynamin-independent, as the authors note, they conclude that dynamin is required for VCC formation based on chemical inhibition or depletion of dynamin 2. However, the dynasore analog may have had off-target effects, and the Dyn2 depletion experiment was only done using the spreading infection approach. To clarify the role of dynamin, the effects of Dyn2 depletion on VCC formation should also be examined using the more readily interpretable VLP addition approach.

Response: We now include Figure 8E-8L, representing VLP addition in MDMs following dynamin 2 knockdown. This intervention significantly diminished VCC formation, as shown in the volume quantitation in Fig 8I.

Part III – Minor Issues: Editorial and Data Presentation Modifications
Please use this section for editorial suggestions as well as relatively minor modifications of existing data that would enhance clarity.

Reviewer #1: Given that the high avidity binding of HIV-1 via Siglec-1 is due to the action of multiple receptors that interact with thousands of sialylated gangliosides, Figure 7C could represent this avidity interaction in the figure showing multiple receptors clustering with several viruses.

Response: We thank the reviewer for this suggestion. In the revised figure (now Figure 9), we show multiple Siglec-1 molecules and particles, although limited in number due to the schematic nature of the diagram.

Reviewer #2: The western blots for inhibition of proteins need to be quantified for at least 3-4 donors (Figure 1F, 2A-B, 5C, ..) and represented as a graph.

Response: We quantified the knockdowns of each cellular protein that was targeted, and include both a representative western blot image and the mean ± SD depletion that was derived from a minimum of 3 experiments. That number is in the text of the Results section for each figure involving siRNA-mediated depletion. We suggest that this number is as meaningful as a single bar graph of depletion would be, especially given the current density of data in the figures.

Why VSVG pseudotyped Vpu-deleted virus is used sometimes (Figure 2)? The use of wt viruses (Bal for instance) would be better or this should be justified.

Response: We initially utilized VSV-G-pseudotyped NLUdel in a few experiments, as this virus forms large VCCs that are enhanced in size because tetherin is not depleted (1). However, we agree with the reviewer that M-tropic viruses such as BaL are more ideal, and the majority of the infection

experiments, including all the new trans-infection data, is performed with BaL. We provide an explanation of this in the text for the experiments where NLUdel is employed.

Figure 1E is complicated to understand, the same for 4I, please explain the legend in another way

Response: We have rewritten the Figure legends as suggested, and hope that the new explanations add clarity for the reader.

Please also provide associated videos to Figure 6

Response: Please note that this Figure shows a time course but utilizes fixed cells for staining of Siglec-1 and for the internalized CD98 antibody, so there is not a video accompanying this experiment.

References:

1. J. E. Hammonds *et al.*, Siglec-1 initiates formation of the virus-containing compartment and enhances macrophage-to-T cell transmission of HIV-1. *PLOS Pathogens* **13**, e1006181 (2017).
